# Quantifying Morphological Change in Stage III Lipedema: A 3D Imaging Study of Population Trends and Individual Treatment Courses

**DOI:** 10.3390/jpm15110525

**Published:** 2025-11-01

**Authors:** Niels A. Sanktjohanser, Nikolaus Thierfelder, Benjamin Beck, Sinan Mert, Benedikt Fuchs, Paul S. Wiggenhauser, Riccardo E. Giunta, Konstantin C. Koban

**Affiliations:** Division of Hand, Plastic and Aesthetic Surgery, LMU University Hospital, LMU Munich, Ziemssenstraße 5, 80336 Munich, Germany; niels.sanktjohanser@med.uni-muenchen.de (N.A.S.); nikolaus.thierfelder@med.uni-muenchen.de (N.T.); benedikt.fuchs@med.uni-muenchen.de (B.F.); severin.wiggenhauser@med.uni-muenchen.de (P.S.W.);

**Keywords:** lipedema, high-volume liposuction, 3D surface imaging, WB360, volumetry, topography

## Abstract

**Background/Objectives**: Lipedema is a chronic disorder characterized by disproportionate fat accumulation in the extremities, causing pain, bruising, and reduced mobility. When conservative therapy fails, liposuction is considered an effective treatment option. Prior studies often relied on subjective or non-standardized measures, limiting precision. This study aimed to objectively assess volumetric changes after liposuction in stage III lipedema using high-resolution 3D imaging to quantify postoperative changes in circumference and volume, providing individualized yet standardized outcome measures aligned with precision medicine. **Methods**: We retrospectively analyzed 66 patients who underwent 161 water-assisted liposuctions (WALs). Pre- and postoperative measurements were performed with the VECTRA© WB360 system, allowing reproducible, anatomically specific quantification of limb volumes and circumferences. Secondary endpoints included in-hospital complications. **Results**: Liposuction achieved significant reductions in all treated regions, most pronounced in the proximal thigh and upper arm. Thigh volume decreased by 4.10–9.25% (q < 0.001), while upper arm volume decreased by 15.63% (left) and 20.15% (right) (q = 0.001). Circumference decreased by up to 5.2% in the thigh (q < 0.001) and 12.27% (q = 0.001) in the upper arm. All changes were calculated relative to baseline values, allowing personalized interpretation of treatment effects. **Conclusions**: This is the first study to objectively quantify postoperative lipedema changes using whole-body 3D surface imaging. By capturing each patient’s contours pre- and postoperatively, this approach enables individualized evaluation while permitting standardized comparison across patients. It offers a precise understanding of surgical outcomes and supports integration of precision medicine principles in lipedema surgery.

## 1. Introduction

Lipedema is a disproportionate fat distribution disorder that almost exclusively affects women and symmetrically involves the extremities [1,2,3]. In addition to functional limitations such as pain, increased bruising tendency and often reduced mobility, the disease also causes esthetic concerns that may result in social stigmatization. Lipedema is divided into three stages; a fourth stage describes the presence of lipolymphedema. The reported prevalence of lipedema varies considerably depending on the studied population. According to the current German S2k guideline, lipedema was diagnosed in 5% of female patients presenting to a general practitioner and in 32.5% of those with leg pain [2,4,5,6]. The exact etiology of the disease remains unknown and is subject to ongoing debate [2,7,8,9].

The basic treatment across all stages of symptomatic lipedema consists of conservative measures, including the consistent use of individually fitted compression garments, regular manual lymphatic drainage, physical activity and a healthy diet [1,2,3]. If conservative therapy is insufficient, liposuction is considered an established surgical option. The primary goal of liposuction in lipedema patients is to achieve a sustained reduction in pain and other symptoms [2,3]. Additionally, liposuction may improve associated orthopedic problems, such as genu valgum or knee joint problems [10]. For many patients, esthetic restoration of limb shape and contour is also of central importance [11,12,13,14]. Given the variability in disease manifestations, liposuction treatment plans are often tailored to the individual, with the number of procedures and target areas adjusted to each patient’s needs and anatomical distribution.

To date, the morphological effects of liposuction have primarily been assessed subjectively by both patients and clinicians. Objective evaluations have mostly relied on tape measurements, which are prone to error and lack reproducibility [15,16,17]. While some earlier studies have employed 3D imaging systems, these were often limited by the technical precision and measurement accuracy available at the time. Technological advances have now led to the development of more accurate 3D imaging systems, enabling highly precise, objective assessment of treatment outcomes [18,19,20,21]. At the time of data collection, patients with stage III lipedema constituted the primary surgical population, as reimbursement by German public health insurance was restricted to this stage under national healthcare regulations [22].

The aim of this study was to objectively assess postoperative changes in limb circumference and volume using an advanced 3D imaging system, which offers significantly greater measurement accuracy than previously available methods. By utilizing this innovative measurement technology, we aimed to generate exact, reproducible volumetric data following liposuction for each patient. In this way, we sought to contribute to a more objective standard in the scientific evaluation of surgical lipedema therapies.

## 2. Materials and Methods

### 2.1. Study Design

This retrospective study aimed to assess changes in limb circumference and volume following liposuction in patients with stage III lipedema. The study was approved by the Ethics Committee of Ludwig-Maximilians-University (LMU) Munich (Project number 24-0252). Informed consent was obtained from all subjects involved in the study.

Data were collected on a patient-specific basis and subsequently pseudonymized for analysis. Data collection was based on standardized pre- and postoperative 3D measurements (creation of 3D avatars) with the VECTRA© WB360 3D surface imaging system (Canfield Sci., Parsippany, NJ, USA) and patient record analysis. All procedures were performed under general anesthesia using WAL with tumescent solution. Due to safety considerations, complete removal of the pathologically altered adipose tissue was not feasible in a single procedure for all documented patients. In accordance with the general guidelines in Germany during the study period, aspirated fat volumes were limited to 8% of body weight per session, necessitating a staged surgical approach. Preoperative data were collected during surgical consultation, while postoperative measurements were taken no earlier than four weeks after surgery to minimize distortion due to postoperative swelling. The exact timing of postoperative scans was not standardized but followed the schedule of routine follow-up visits, resulting in heterogeneous imaging intervals across patients. For each operation, outcome assessment was based on a single preoperative and a single postoperative 3D scan. All measurements followed a standardized protocol.

Secondary outcome measures included the occurrence of treatment-related adverse events during hospitalization to assess the frequency of these complications. Patient comorbidities were recorded as well. Data were extracted from patient records.

### 2.2. Study Population

**Figure 1:**
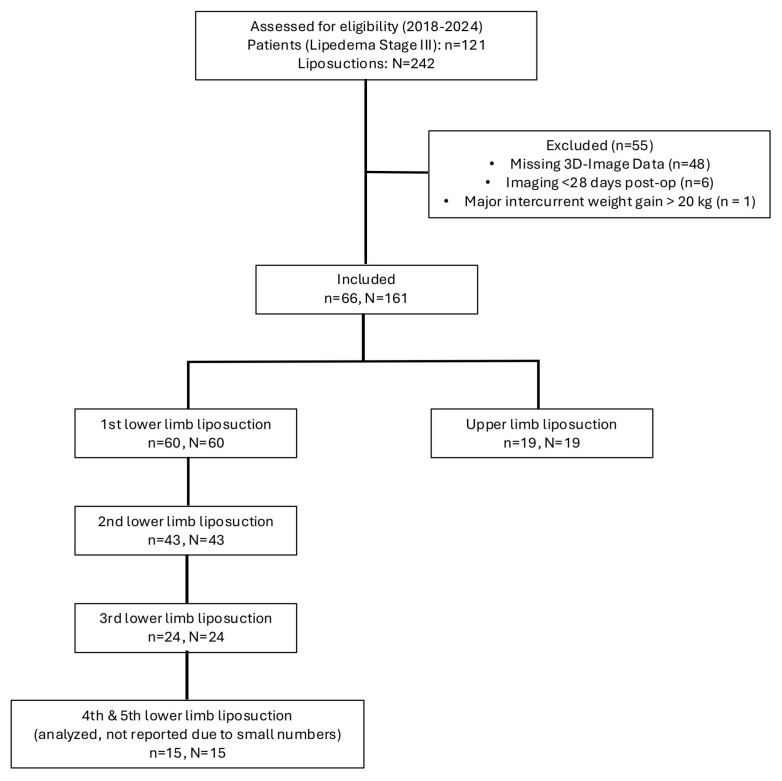
Depiction of the recruitment process of patients with stage III lipedema. Of the initially screened 121 patients with 242 documented liposuctions, 55 patients were excluded due to missing 3D imaging data or insufficient therapy adherence. The final study cohort comprised 66 patients undergoing 161 liposuction procedures, which were included in the analysis.

The recruitment and exclusion process is illustrated in Figure 1.

#### 2.2.1. Inclusion Criteria

Patients were eligible if they had undergone both conservative therapy for at least six months and at least one liposuction procedure for stage III lipedema between 16 November 2018, and 20 June 2024, at the surgical clinic of the LMU Munich University Hospital. Consistent adherence to conservative treatment, especially the sustained use of compression garments, was an additional inclusion criterion.

#### 2.2.2. Exclusion Criteria

Patients were excluded for incomplete data, poor therapy adherence, or loss to follow-up.

### 2.3. Data Collection and Measurement of Circumference and Volume

A VECTRA© WB360 camera (Canfield Scientific GmbH, Parsippany, NJ, USA) was used to generate 3D avatars. This system uses 92 individual cameras to capture a full-body image within 3.5 milliseconds, which is then reconstructed into a topographical 3D model. Patients stood in a standardized position on marked floor points.

Secondary outcome measures included the assessment of treatment-related adverse events during hospitalization such as clinically detectable infections, transfusion-requiring blood loss, venous thromboembolism and pulmonary embolism. Perioperative care followed institutional routines to minimize blood loss (e.g., preoperative coagulation assessment and management of antithrombotic therapy, epinephrine-containing tumescent infiltration, meticulous postoperative hemostasis with immediate compression, and postoperative thromboprophylaxis and clinical monitoring). Intraoperative cell salvage (cell saver) was not part of routine care in this cohort. Detailed intraoperative metrics (e.g., aspirate volume, perioperative hemoglobin/hematocrit trajectories) were not uniformly documented across the study period and were therefore not analyzed to avoid information bias. Safety outcomes were pre-specified as exploratory and are presented in a descriptive manner.

### 2.4. Measurement Protocol

#### 2.4.1. Lower Limb Measurements

**Figure 2:**
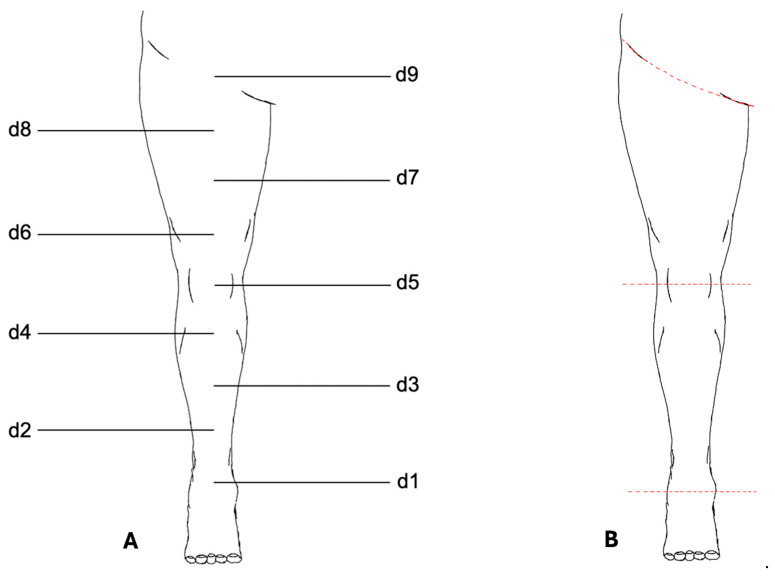
Measurement protocol for the lower limb. (**A**) Schematic illustration of the standardized measurement points (d1–d9) defined along the central axis of the lower limb. Circumference measurements were performed at d1–d8; d9 was used only as an anatomical reference point. (**B**) Digital segmentation of the lower limb using VECTRA© software. The leg was separated proximally at the inguinal ligament and distally at d1. Additional segmentation at the patella midpoint (d5) allowed for independent measurement of thigh and lower leg volumes. Colored lines indicate the segmentation planes.

The VECTRA© software application (version 7.3.2) was used for measurement analysis (Figure 2). To standardize the placement of measurement points, a central axis of the lower limb was first defined using two anatomical reference points: proximally the midpoint of the inguinal ligament, and distally the midpoint of the line connecting the medial and lateral malleoli.

A third anatomical reference point was placed on this axis at the midpoint of the patella. The distances between the proximal and middle reference points and between the middle and distal reference points were then divided into four equal segments, resulting in a total of nine measurement points along the axis. These points were numbered from distal to proximal as follows: d1, d2, …, d9.

Standardized circumference measurements were performed at eight measurement points (d1–d8). The proximal reference point (d9) above the inguinal ligament was not included in circumference measurements and served only to define the axis of the leg.

For volume determination, the leg was digitally separated from the body trunk at the inguinal ligament using the VECTRA© software and the foot was separated at point d1. Further segmentation at the midpoint of the patella (d5) allowed for the independent measurements of the thigh and lower leg.

#### 2.4.2. Upper Limb Measurements

**Figure 3:**
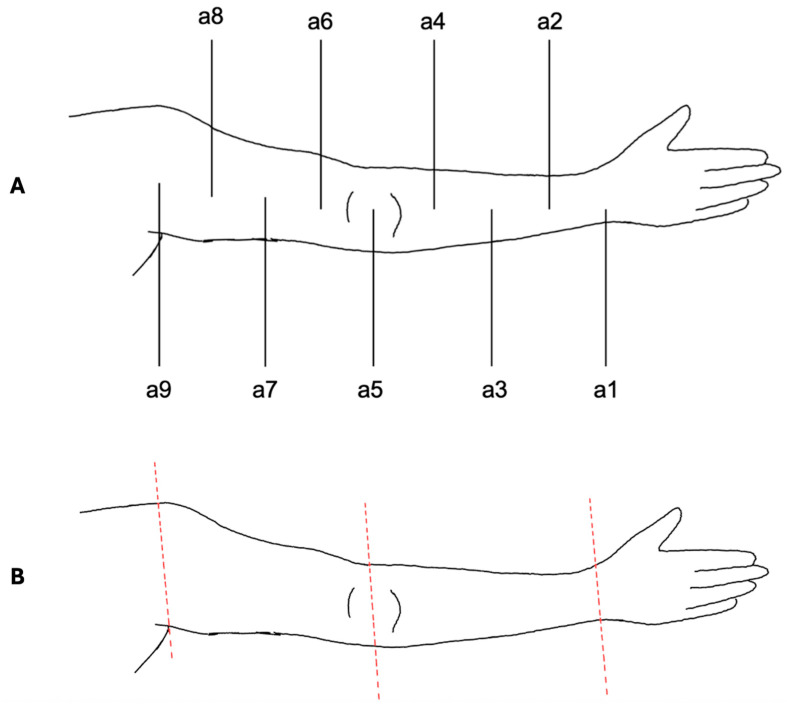
Measurement protocol for the upper limb. (**A**) Schematic illustration of the standardized measurement points (a1–a9) defined between acromion (proximal reference), olecranon (middle reference), and wrist (distal reference). Circumference measurements were performed at a1–a8; a9 served only for alignment. (**B**) Digital segmentation of the upper limb using VECTRA© software. The arm was separated proximally at a9 and distally at a1. Segmentation at the elbow level (a5) enabled independent assessment of upper and lower arm volumes. Colored lines indicate the segmentation planes.

The VECTRA© software was also used for upper limb measurements (Figure 3). To ensure standardization, the acromion served as the proximal reference point, the olecranon as the middle reference point and the midpoint of the line connecting the styloid process of the ulna and radius (Restricta) as the distal reference point.

Measurements followed the same protocol as for the lower limbs. To differentiate between measurement points, the nomenclature was established as follows: a1, a2, …, a9. Similarly to the lower limb, point a9 was used only for alignment and was not included in the actual measurements.

Volume segmentation was performed by digitally separating the arm at a9 from the body trunk and the hand at a1. The distinction between the upper and lower arm was achieved by segmentation at a5 at the elbow.

For both lower and upper limb analyses, a central anatomical axis was defined by connecting reproducible skeletal landmarks. For the legs, the axis extended from the midpoint between the medial and lateral malleoli to the midpoint of the inguinal ligament. For the arms, the axis was defined from the acromion to the midpoint of the line connecting the ulnar and radial styloid processes (Restricta). Segmentation planes were created orthogonally to this axis at predefined anatomical levels: the inguinal ligament served as the proximal cut-off for separation of the lower limb from the trunk, while the malleolar level defined the distal end. For the arms, the acromion plane separated the upper limb from the trunk, and the wrist plane (Restricta midpoint) defined the distal boundary. Additional segmentation at the patella midpoint (legs) and olecranon (arms) enabled separate evaluation of proximal and distal compartments. Surface reconstruction and smoothing of the 3D avatars were performed automatically by the VECTRA^®^ WB360 software using standardized algorithms for mesh generation. No user-adjustable thresholds were applied. This workflow minimized operator dependence and ensured a standardized, reproducible pipeline for all measurements. For a more comprehensive visualization of the segmentation workflow, including anatomical axis construction, auxiliary landmarks, and orthogonal plane definitions, an extended schematic overview is provided in the Supplementary Material (Supplementary File S1). This document illustrates the geometric consistency and reproducibility of the segmentation model applied across all 3D analyses.

#### 2.4.3. Data Analysis and Statistics

Measurement results were pseudonymized, organized in a database and prepared for statistical analysis. Pre- and postoperative values were compared pairwise for each patient, with differences calculated in both absolute and percentage terms. Missing or incomplete datasets were excluded from the analysis.

Statistical analysis of circumference and volume measurements was performed using SPSS (IBM© SPSS Statistics 29.0). Normality was assessed with the Shapiro–Wilk test. Due to non-normal distributions, the Wilcoxon signed-rank test was applied. To address multiple testing, *p*-values were adjusted using the Benjamini–Hochberg false discovery rate (FDR) procedure, with q < 0.05 considered significant. Confidence intervals (95% CI) were calculated in RStudio (Version 2025.09.0+387; Posit Software©) to assess the robustness of the effects. For clinical interpretability, mean changes are reported in the text. Confidence intervals are based on Wilcoxon signed-rank tests and therefore reflect median changes.

## 3. Results

A total of 66 patients with stage III lipedema who underwent 161 documented WAL procedures were included in this study. The mean age at the time of the first procedure was 44.7 ± 12.55 years (range: 21–74 years). Postoperative 3D measurements were obtained at a median of 50 days overall (IQR 41–110). Timing varied across procedures: After the first leg surgery, scans were performed at a median of 47 days (IQR 41–89, after the second procedure at 61 days (IQR 41–126) and after the third procedure at 54 days (IQR 43–102). Postoperative imaging after arm surgery was obtained at a median of 49 days (IQR 33–81).

At the beginning of treatment, prior to the first liposuction, the mean body mass index (BMI) of the study population was 34.89 ± 4.27 kg/m^2^ (range: 26.9–48.19 kg/m^2^), and the average body weight was 95.79 ± 13.04 kg (range: 68–136 kg). With subsequent procedures, a downward trend in BMI was observed. The mean BMI decreased to 34.35 ± 4.11 kg/m^2^ before the second surgery and further to 33.48 ± 4.22 kg/m^2^ before the third.

To assess potential perioperative risks, documented risk evaluations conducted by anesthesiologists during the preoperative consultation and physical examination were reviewed. These assessments, based on standardized anamnesis and clinical findings, included both known pre-existing conditions and factors deemed relevant for perioperative management (e.g., depression, asthma, prolonged menstrual bleeding, etc.). Overall, 86.36% of patients presented with at least one comorbidity relevant to perioperative risk assessment. The identified risk factors and comorbidities are summarized in the following table (Table 1).

To account for interindividual differences in body composition and baseline measurements, all reductions in volume and circumference are expressed as percentage decreases relative to the respective preoperative values. This approach allows for standardized comparisons across patients with varying body sizes and ensures that the reported outcomes are proportional and reflective of each individual’s unique anatomical baseline. In the following analysis, surgeries of the lower and upper limbs are considered separately. Most patients initially underwent liposuction of the legs, followed by a subsequent procedure on the arms. However, in some cases, this exact sequence was not strictly followed.

All calculated 95% confidence intervals for every measurement point are provided in detail in the Supplementary Data (Figures S1–S4). In the following Results section, we focus on the most pronounced and clinically relevant effects, while representative q-values and selected confidence intervals are highlighted in the text for clarity.

### 3.1. Lower Limbs—Changes in Circumference and Volume

#### 3.1.1. First Surgery

The first procedure resulted in significant volume reductions across nearly all examined areas. For the lower leg, the mean volume reduction was 4.32% (right) and 4.10% (left) (q < 0.001; right: 95% CI −6.04 to −2.55; left: 95% CI −5.57 to −2.35), whereas the thigh showed more pronounced reductions of 8.66% (right) and 9.25% (left) (q < 0.001; right: 95% CI −11.33 to −6.04; left: 95% CI −11.25 to −6.22).

**Figure 4:**
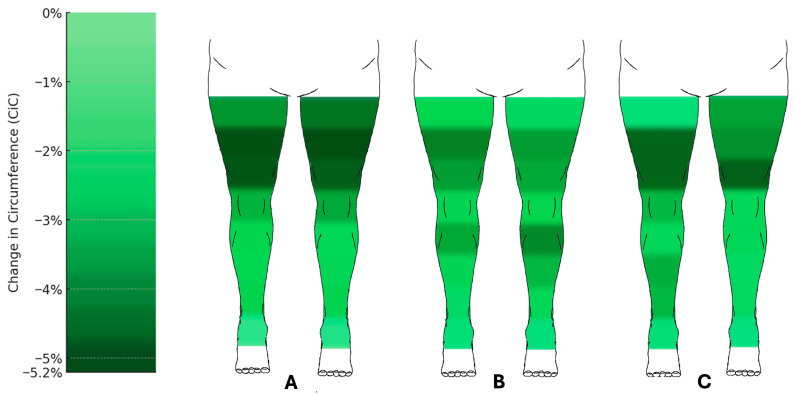
Changes in circumference of the lower limbs following liposuction. Heat map representation of mean percentage change in circumference (CiC) across measurement points after (**A**) the first (n = 60), (**B**) the second (n = 43), and (**C**) the third (n = 24) liposuction procedures. Reductions were most pronounced at proximal thigh levels (up to −5.2%), whereas distal measurement points showed little or no change. Median change in circumference relative to preoperative baseline (%) with 95% confidence intervals. Comprehensive CI tables for all measurement points are provided in the Supplementary Data (Figures S1–S3).

Circumference measurements also showed marked reductions (Figure 4). At the lower leg, the circumferences at the distal measurement point d1 showed no significant changes. However, measurement points d2 to d4 showed significant reductions, with d3 decreasing by an average of 2.50% (right) and 2.41% (left) (q < 0.001; right: 95% CI −3.99 to −1.75; left: 95% CI −4.31 to −1.43). The most pronounced reductions were observed at the proximal measurement point d7, with circumference decreases of 5.15% (right) and 5.20% (left) (q < 0.001; right: 95% CI −6.54 to −3.59; left: 95% CI −6.63 to −3.8).

#### 3.1.2. Second Surgery

Volume reductions continued after the second procedure.

The lower leg volume decreased by 4.77% (right) and 5.66% (left) (right: q = 0.005, 95% CI −7.15 to −2.39; left: q = 0.009, 95% CI −8.37 to −1.85), while the thigh showed reductions of 7.14% (right) and 5.68% (left) (right: q = 0.001, 95% CI −9.89 to −4.59; left: q = 0.002, 95% CI −9.56 to −3.35).

Circumference measurements at the lower extremities also demonstrated significant reductions (Figure 3). Measurement points d2 to d4 showed notable changes, with d3 decreasing by 2.37% (right) and 3.23% (left) (right: q = 0.018, 95% CI −4.07 to −0.58; left: q = 0.004, 95% CI −5.7 to −1.49), and d4 by 3.57% (right) and 4.12% (left) (q = 0.001; right: 95% CI −4.86 to −2.05; left: 95% CI −6.14 to −2.39). The most significant reductions at the thigh were observed at d7, with decreases of 4.26% (right) and 3.73% (left) (q = 0.001; right: 95% CI −6.28 to −2.3; left: 95% CI −5.89 to −2.15).

#### 3.1.3. Third Surgery

The third procedure led to further mean volume reductions. The lower leg showed volume reductions of 5.07% (right) and 3.55% (left) (right: q = 0.023, 95% CI −8.85 to −1.31; left: q = 0.026, 95% CI −6.79 to −0.56), while the thigh showed moderate reductions of 3.25% (right) and 7.24% (left) (right: q = 0.173, 95% CI −8.24 to 1.99; left: q = 0.017, 95% CI −12.24 to −2.75, respectively).

Circumference measurements also demonstrated significant changes (Figure 3).

At the lower leg, the circumferences at measurement points d1 to d4 decreased, with the most significant reduction at d2, showing decreases of 3.24% (right) and 1.97% (left) (right: q = 0.024, 95% CI −5.52 to −0.69; left: q = 0.173, 95% CI −4.6 to 0.61, respectively). At the thigh, the most pronounced reductions were observed at d7, with decreases of 4.80% (right) and 3.97% (left) (q = 0.017; right: 95% CI −7.37 to −2.04; left: 95% CI −6.24 to −1.51).

#### 3.1.4. Fourth and Fifth Surgeries

Volume and circumference reductions were also observed after four and five surgical procedures. However, due to the limited number of documented patients in these groups, no statistically significant changes were detected.

### 3.2. Upper Limbs—Changes in Circumference and Volume

**Figure 5:**
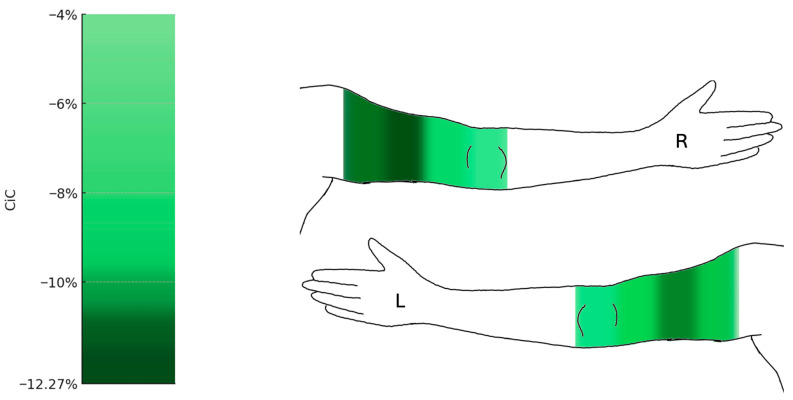
Changes in circumference of the upper limbs following liposuction (n = 19). Heat map representation of mean percentage change in circumference (CiC) of the upper extremities. Significant reductions were observed in the proximal upper arm segments (up to −12.27%), while the forearm and wrist (a1–a4) remained largely unchanged. Median change in circumference relative to preoperative baseline (%) with 95% confidence intervals. Comprehensive CI tables for all measurement points are provided in the Supplementary Data (Figure S4). L = left arm; R = right arm.

No significant reductions in volume were observed in the forearm, with decreases of 3.21% (right) and 4.98% (left). In contrast, the upper arm showed significant reductions of 20.15% (right) and 15.63% (left) (q = 0.001; right: 95% CI −25.5 to −11.29; left: 95% CI −22.71 to −9.57). Circumference measurements demonstrated predominantly significant reductions (Figure 5).

At the upper extremities, circumferences at the distal measurement point a1 remained statistically unchanged, while the forearm showed noticeable changes at measurement points a2 to a4, though none reached statistical significance. The upper arm showed the most pronounced reductions at the proximal measurement points, with a decrease of 12.27% (right) and 11.59% (left) at a7 (q = 0.001; right: 95% CI −24.46 to −10.03; left: 95% CI −15.62 to −6.4), and 11.84% (right) and 10.77% (left) at a8 (q = 0.001; right: 95% CI −17.61 to −5.41; left: 95% CI −16.83 to −6.29).

### 3.3. Incidence of Infection and Transfusion Requirement

Among the 161 surgical procedures, two cases of clinically detectable postoperative infection were recorded (1.24%), while transfusion-requiring blood loss occurred in 13 cases (8.07%). No cases of deep vein thrombosis or pulmonary embolism were observed during the hospital stay.

## 4. Discussion

This study is the first to precisely and objectively assess volumetric and circumferential changes in the limbs following WAL in patients with stage III lipedema using a 3D whole-body surface imaging system. While previous research on the effectiveness of liposuction has primarily relied on tape measurements, these methods are prone to errors due to variability in tension, individual measurement inaccuracies, and limited reproducibility [15,16,17]. Alternatively, 3D surface imaging systems have been used in previous studies; however, these studies are relatively dated and relied on devices that reflected the technical standards of their time, which were significantly less precise compared to modern systems. In contrast, our study employs high-resolution 3D surface imaging to analyze postoperative changes with enhanced precision and reliability. Utilizing the VECTRA© WB360 system allows for standardized, reproducible measurement of limb circumference and volume, thus elevating the methodological standard in evaluating the morphological effects of surgical treatment of lipedema. The system’s accuracy and efficiency have been previously validated by Xu et al., supporting its use in clinical research [23].

Additionally, expressing circumferential and volumetric changes in relative percentage reductions compared to baseline represents a methodological advancement. While previous studies primarily reported absolute values, our approach allows for improved comparability among patients with different body compositions [16,24], thereby aligning outcome evaluation with a more personalized patient care perspective.

To address the possibility that the repeated significant findings may be attributed to weight fluctuations between procedures, it is important to consider the BMI progression across the successive lower limb liposuctions. The average BMI prior to the first procedure was 34.89 ± 4.27 kg/m^2^, followed by 34.35 ± 4.11 kg/m^2^ before the second, and 33.48 ± 4.22 kg/m^2^ before the third. These minor changes indicate that patients largely maintained stable body weight during the treatment period. The slight decrease may reflect the cumulative removal of adipose tissue, but should not be interpreted as conventional weight loss, as liposuction is not intended for that purpose, but rather to relieve symptoms.

This is supported by Baumgartner et al. [14], who found that average body weight twelve years after liposuction had slightly increased, showing no relevant long-term change. Their findings reinforce that liposuction is not a weight-loss procedure but primarily serves to reduce symptoms. Their study, however, examined long-term outcomes, while our data reflect a shorter observation period.

The significant reductions in volume and circumference across all treated body regions illustrate the measurable volumetric and circumferential changes achieved with the WAL procedure. Given the characteristic fat distribution in lipedema [2], the most pronounced changes were expected at the proximal measurement points of the upper and lower limbs, whereas distal points such as d1 (ankle) and a1 (wrist) were anticipated to remain largely unchanged. This was confirmed by our measurements, reflecting anatomical characteristics and the targeted impact of WAL in these areas. Forearm changes were small and remained below statistical significance, indicating only a modest tendency compared with the pronounced proximal reductions. This pattern is consistent with the proximal predominance of arm involvement in lipedema and with operative prioritization of proximal compartments [25], plausibly explaining the limited distal response without suggesting a measurement artifact. While some asymmetries between left and right limb measurement reductions were observed, particularly in later procedures with reduced sample sizes, these differences were small and may reflect technical factors such as aspiration volume or patient positioning. A formal analysis of side-to-side variation was not within the scope of this study. The absence of significant changes at distal measurement points further underscores the precision of our measurement methodology, as these regions physiologically lack pronounced fat accumulations.

Schmeller et al. (2010) [17] reported an average reduction in proximal thigh circumference of 8 cm and a mean reduction of 4 cm in the lower leg over a follow-up period of 3 years and 8 months post-liposuction. Additionally, most patients experienced a decrease in clothing size, highlighting the procedure’s impact on body contouring. However, Schmeller et al. presented only absolute values, which do not account for interindividual differences in body size and composition. In contrast, our study also demonstrated significant reductions in leg circumferences, but we expressed our results in percentage reductions to provide a more standardized and comparable measure that accounts for individual variations in body proportions.

Rapprich et al. (2011) observed an average leg volume reduction of 6.99% six months after liposuction, further reinforcing the efficacy of WAL as a treatment for lipedema [15]. In their study, volume measurements were obtained using a Bodytronic© system by Bauerfeind© (Bauerfeind AG, Zeulenroda-Triebes, Germany), which, while innovative at the time, offers lower spatial resolution compared to current high-resolution imaging technologies. In our study, we calculated comparable volume reductions for the first liposuction treatment of the lower limbs, thereby confirming and further substantiating the findings of Rapprich et al. [15]. A key distinction, however, is that our study utilized a more advanced 3D camera system, enabling a higher-resolution and more precise analysis of postoperative volume changes. Moreover, while the Bodytronic© system relies on a turntable-based scanning process that requires the patient to remain still for a longer duration—making it more susceptible to motion artifacts and distortion of the 3D avatar—our VECTRA© WB360 system captures the entire body surface within a fraction of a second. This not only improves image resolution but also minimizes the risk of movement-related inaccuracies, thereby enhancing the overall reliability and validity of the measurements. Additionally, while Rapprich et al. [15] focused on the effects of a single liposuction procedure, our study provides the advantage of assessing multiple liposuction procedures per patient, allowing for a more comprehensive evaluation of the effects of WAL.

Our in-hospital complication rate of 1.24% for postoperative infections is consistent with previous studies, which reported rates between 0.34% and 1.79% [16,24,26,27]. However, it should be noted that the patient cohorts in these studies were, in some cases, less obese (e.g., Schmeller et al. [16] reported a mean body weight of 79.3 kg [range: 50–123 kg]) and had fewer pre-existing conditions than our study population. This finding reinforces the overall safety of the procedure, even in a more complex patient cohort.

In contrast, the rate of transfusion-requiring blood loss in our study was higher than previously reported. A possible explanation for this is that our patient cohort had a particularly high burden of comorbidities, with 86.36% of patients affected. Furthermore, unlike previous studies that included patients across all lipedema stages [16,24,27], our study exclusively investigated patients with stage 3 lipedema, a more advanced form of the disease often associated with increased tissue fragility [2,28] and dilated capillaries [29]. This factor may have contributed to a heightened bleeding tendency, thereby necessitating a higher rate of transfusions. Given the high transfusion rate, more detailed preoperative lab screening, especially of iron status and coagulation, may be warranted in selected patients. This should be evaluated in future prospective studies.

The observed transfusion rate should be interpreted within the study’s exploratory safety scope and the cohort characteristics. Because detailed intraoperative metrics (e.g., aspirate volumes, perioperative hemoglobin trajectories) were not uniformly documented retrospectively, we refrained from potentially biased subgroup analyses. The present manuscript prioritizes objective 3D outcomes as its primary contribution. Ongoing prospective work applies predefined perioperative variables to enable granular risk profiling and clinical management recommendations.

The strength of this study lies in its precise and standardized measurement methodology, using 3D avatars to enable accurate and reproducible quantification of postoperative changes after WAL. While the study provides robust volumetric and circumferential outcome data, several methodological aspects need to be acknowledged. First, the retrospective design inherently limits control over confounding variables and reduced statistical power in subgroups with more than three procedures. In addition, the analysis was restricted to stage III patients within the German reimbursement framework. Nevertheless, the consistent proximal–distal reduction patterns observed are anatomically determined and therefore likely relevant across disease stages and healthcare systems, although confirmation in broader prospective studies is warranted.

Second, comparator measurements such as protocolized tape assessments were not part of routine care and were therefore unavailable for validation within the same schema or timepoints. A post hoc validation subset was thus not feasible. Furthermore, follow-up intervals could not be standardized, as imaging was performed during routine postoperative visits rather than at fixed timepoints. Although analyses were restricted to scans obtained ≥ 28 days after surgery to limit early edema bias, heterogeneity remained. A sensitivity analysis restricted to scans > 90 days showed consistent trends, but significance was attenuated due to the markedly reduced sample size (Supplementary File S2).

Third, body weight and BMI were only recorded preoperatively during surgical preparation, precluding longitudinal adjustment of outcomes for weight changes. The available data across successive procedures showed only a slight downward trend in BMI, suggesting overall stable weight during the observation period. Consequently, the observed volumetric reductions are unlikely to reflect conventional weight loss but rather the local effects of liposuction.

Fourth, certain constraints apply to the imaging workflow. Segmentation planes were consistently defined at reproducible anatomical landmarks (inguinal ligament, patella midpoint, and malleoli for the lower limbs; acromion, olecranon, and styloid processes for the upper limbs). Surface reconstruction and smoothing were performed automatically by the VECTRA^®^ WB360 software using standardized algorithms without user-adjustable thresholds. While this ensured a standardized and operator-independent pipeline, it also precluded investigator-driven optimization. Although external studies have demonstrated high reproducibility of the WB360 system, we did not perform a formal intra-/inter-rater or test–retest reliability analysis within our workflow. This should be addressed in future prospective research.

Finally, patient-reported outcomes such as pain or quality of life were not systematically collected in this retrospective dataset. Prospective studies currently underway in our department are designed to address this gap by combining 3D volumetry with validated patient-reported outcome measures, thereby linking morphological change more directly with clinical benefit.

Despite this, the study represents a methodological advancement, as the advanced 3D imaging system provides unprecedented accuracy in assessing postoperative outcomes. Beyond its value for standardized outcome research, the applied 3D surface imaging system offers a powerful tool for individualized treatment monitoring in clinical practice, particularly after staged liposuction procedures, enabling both objective therapeutic evaluation and transparent, patient-accessible visualization of surgical results. Previous studies by Schmeller et al. [16] and Rapprich et al. [15] demonstrated that postoperative reductions in limb volume after liposuction were accompanied by significant improvements in pain, mobility, and overall quality of life. Although the present analysis focused on morphological outcomes, it is plausible that the volumetric reductions observed in our cohort similarly translate into symptomatic relief, as suggested by these earlier reports. This study contributes to the scientific evaluation of WAL in lipedema treatment and lays the groundwork for future prospective studies to validate findings, assess long-term outcomes, and further explore efficacy, safety, and potential complications.

## 5. Conclusions

This study demonstrates that water-assisted liposuction in stage III lipedema achieves significant reductions in limb volume and circumference when measured with high-resolution 3D surface imaging. By using objective, standardized, and reproducible methods instead of subjective or tape-based assessments, we provide robust data that strengthen the evidence base for surgical therapy in lipedema. Expressing results as relative percentage changes to each patient’s baseline allows for individualized interpretation of treatment effects while maintaining comparability across patients, thereby reflecting key principles of precision medicine and personalized therapy evaluation. Integrating advanced imaging technologies into surgical outcome assessment enhances transparency, supports patient communication, and aligns lipedema treatment with the broader goals of evidence-based medicine and personalized medicine in clinical practice. Within the context of multidisciplinary therapeutic strategies, our findings highlight the potential of modern imaging to bridge clinical care and scientific evaluation, contributing to more precise, patient-centered management of lipedema.

## Figures and Tables

**Table 1:** Perioperative risk factors and comorbidities values are presented as n (%). Patients could present with more than one comorbidity, therefore percentages do not sum to 100%.

Perioperative Risk Factors and Comorbidities	n (%)
Musculoskeletal and Neuropsychiatric	27 (40.91%)
Gastrointestinal	23 (38.33%)
*Bariatric Surgery*	12 (18.18%)
Thyroid	21 (31.82%)
Respiratory	15 (22.73%)
Cardiovascular	16 (24.24%)
Coagulation	13 (19.70%)
*Procoagulant*	4 (6.06%)
*Anticoagulant*	9 (13.64%)
Patients with ≥1 risk factor or comorbidity	57 (86.36%)

## Data Availability

The original contributions presented in this study are included in the article/Supplementary Material. Further inquiries can be directed to the corresponding author(s).

## Supplementary Materials

The following supporting information can be downloaded at: https://www.mdpi.com/article/10.3390/jpm15110525/s1, File S1: Segmentation Model, File S2: Sensitivity Analysis _90days; Figure S1: First Lower Limb Liposuction; Figure S2: Second Lower Limb Liposuction; Figure S3: Third Lower Limb Liposuction; Figure S4: Upper Limb Liposuction.
