# Supplementary material for: Quantifying Morphological Change in Stage III Lipedema: A 3D Imaging Study of Population Trends and Individual Treatment Courses
Source: J Pers Med. 2025 Nov 1;15(11):525. doi: 10.3390/jpm15110525 (PMC12653540; doi:10.3390/jpm15110525)

Segmentation model

Schematic illustration of the anatomical axis and segmentation planes as described in the main manuscript. Auxiliary landmarks (e.g., acromion, olecranon, patella, and malleoli) were used to define reproducible central axes, with orthogonal planes dividing each limb into proximal and distal compartments. This visualization serves as an example segmentation mask illustrating the standardized workflow applied across all measurements.

- Auxiliary points defined by anatomical landmarks for constructing central axes
- Construction guidelines used for geometric axis alignment and segment definition
- Primary measurement points used for circumference and volume quantification
- Anatomical axes or segmentation planes used for volumetric division

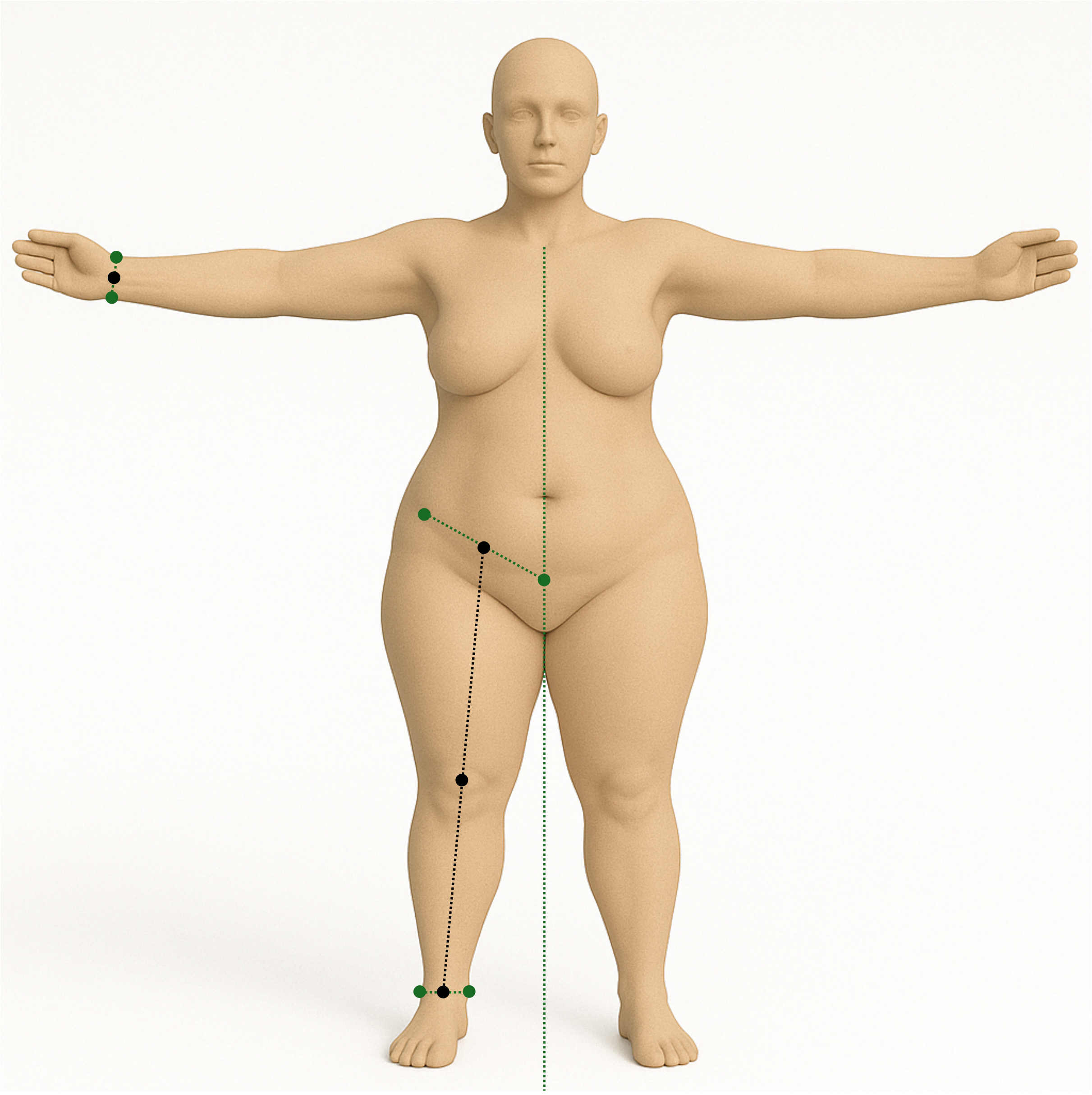

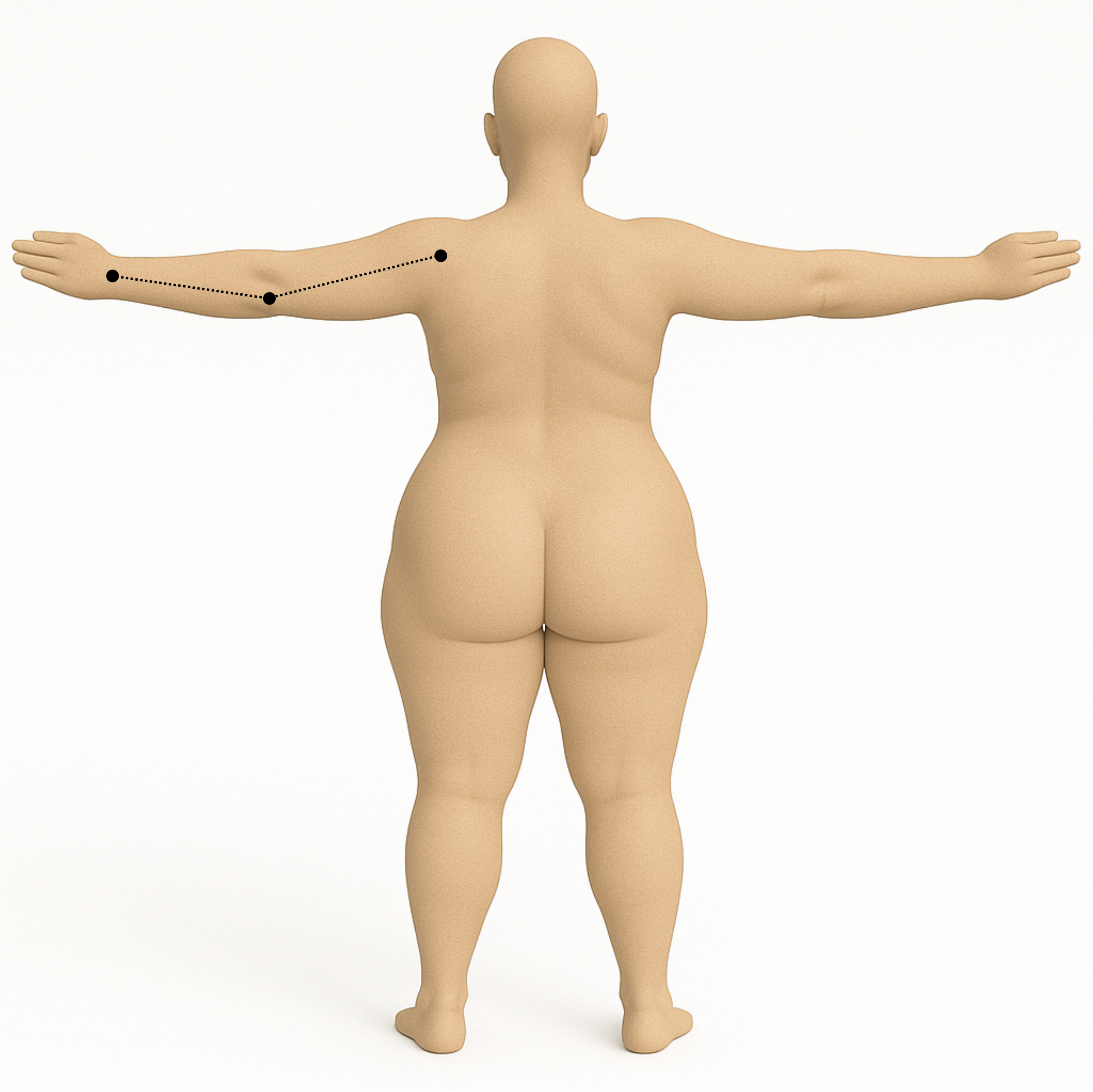

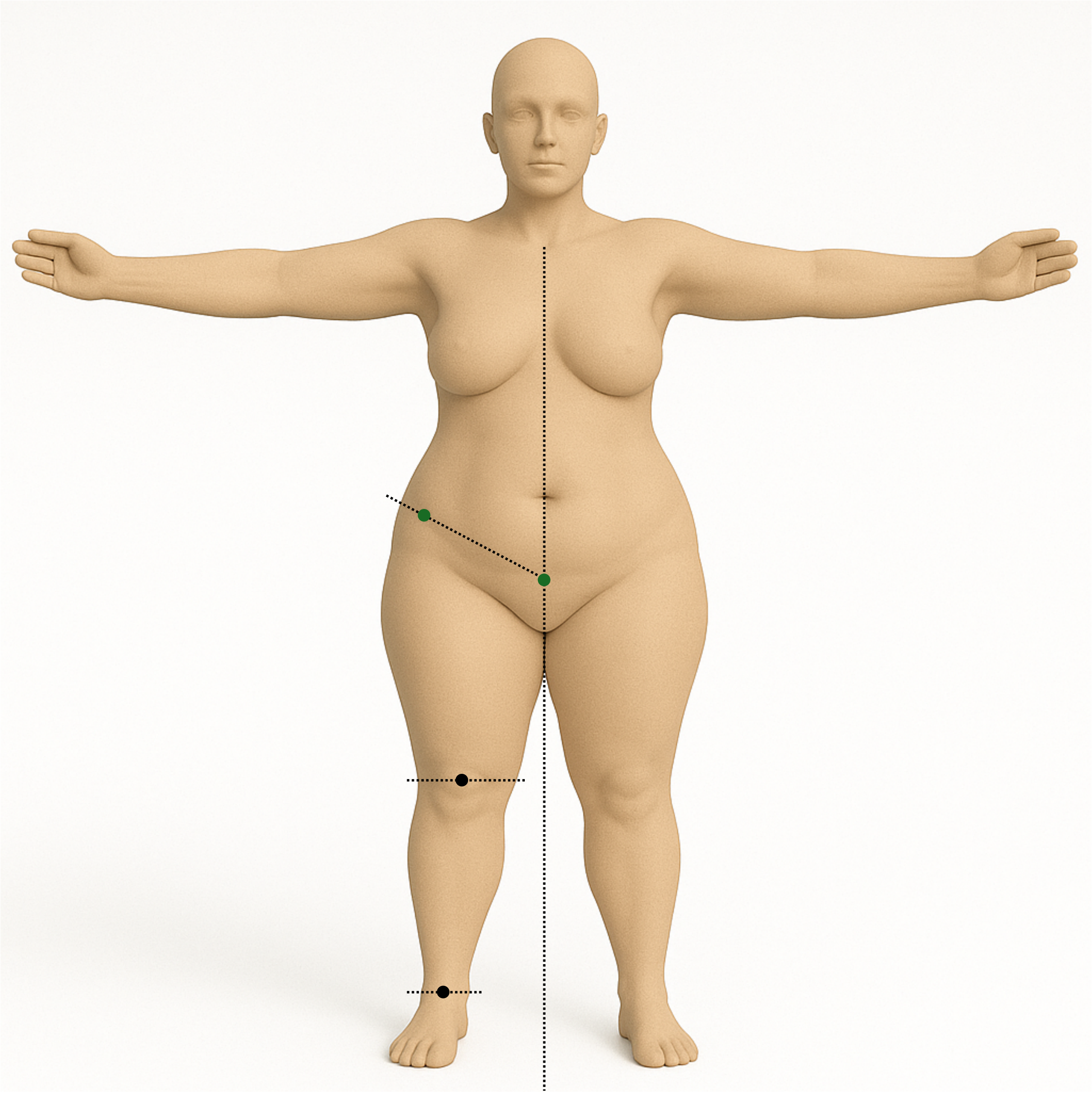

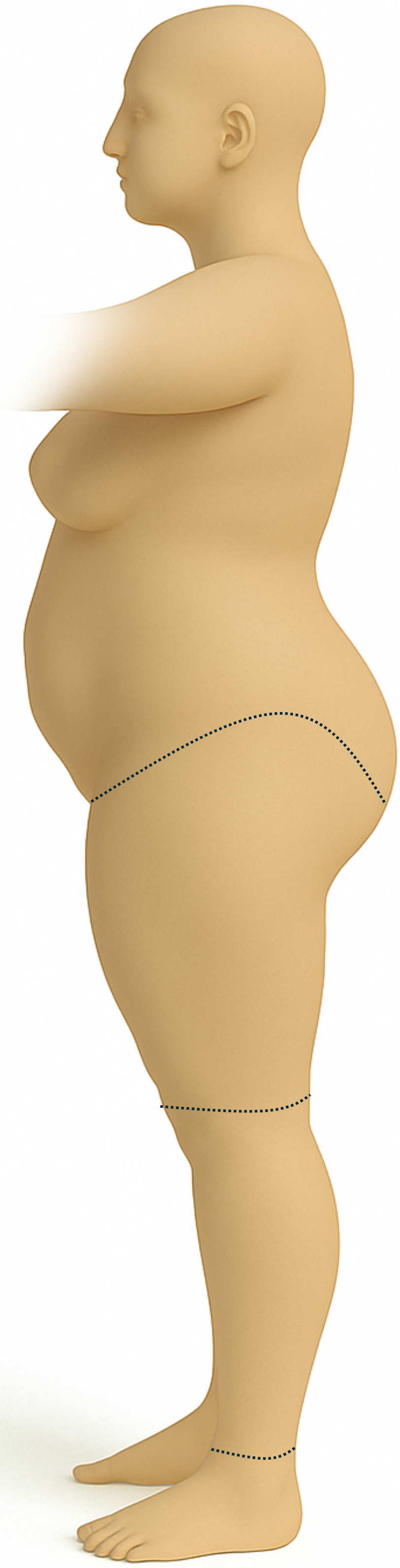

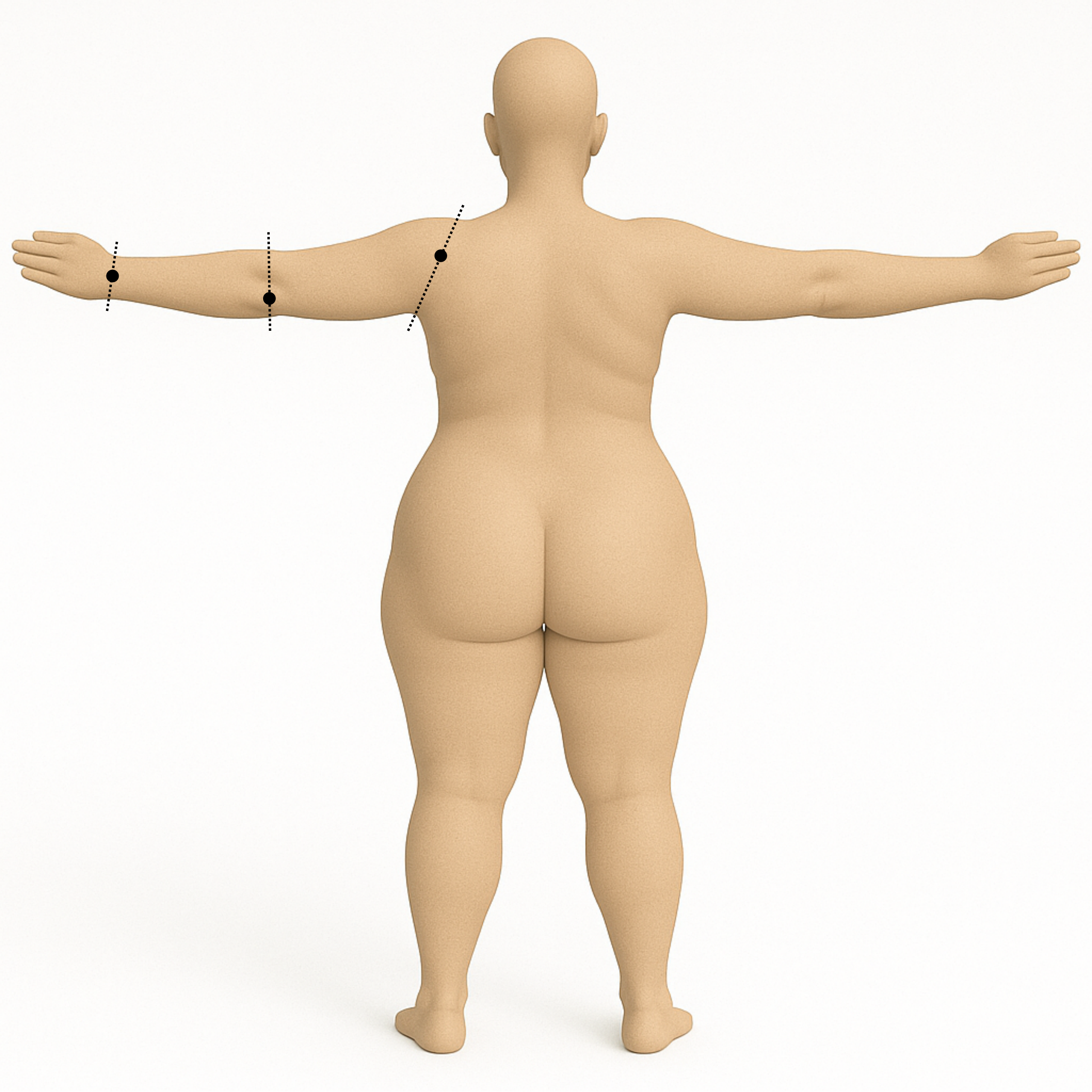

Supplement: Supplementary file 1 [file jpm-15-00525-s001.zip › File S1_Segmentation Model.pdf]
