# Supplementary material for: Quantifying Morphological Change in Stage III Lipedema: A 3D Imaging Study of Population Trends and Individual Treatment Courses
Source: J Pers Med. 2025 Nov 1;15(11):525. doi: 10.3390/jpm15110525 (PMC12653540; doi:10.3390/jpm15110525)
Supplement: Supplementary file 1 [file jpm-15-00525-s001.zip › File S2_Sensitivity Analysis _90days.pdf]

Sensitivity Analysis (>90 Days Post-Operative)

To minimize potential bias from early postoperative edema, a sensitivity analysis was performed including only procedures with 3D imaging obtained more than 90 days after surgery. The direction and magnitude of changes remained consistent with the main analysis, although statistical significance was attenuated due to the markedly smaller subgroup size. In particular, the number of postoperative scans of the upper limbs was limited, resulting in non-significant but directionally concordant q-values in this region.

Lower Limbs

Measurement Region	Mean % Decrease	Q (FDR-adjusted)
V T r	6.78	0.003
V T l	7.06	0.002
V LL r	4.49	0.005
V LL l	4.68	0.004
d1 r	0.83	0.394
d1 l	0.53	0.394
d2 r	2.14	0.07
d2 l	2.45	0.049
d3 r	1.94	0.052
d3 l	2.33	0.017
d4 r	3.17	0.002
d4 l	2.96	0.002
d5 r	3.74	0.004
d5 l	3.52	0.003
d6 r	4.91	<0.001
d6 l	4.53	<0.001
d7 r	4.46	0.002
d7 l	4.24	0.002
d8 r	2.6	0.027
d8 l	2.36	0.067

Upper Limbs

Measurement Region	Mean % Decrease	Q (FDR-adjusted)
V UA r	14.13	0.085
V UA l	16.89	0.139
V LA r	n/a	n/a
V LA l	n/a	n/a
a1 r	n/a	n/a
a1 l	n/a	n/a
a2 r	n/a	n/a
a2 l	n/a	n/a
a3 r	n/a	n/a
a3 l	n/a	n/a
a4 r	n/a	n/a
a4 l	n/a	n/a
a5 r	1.03	0.335
a5 l	2.18	0.139
a6 r	6.6	0.085
a6 l	7.33	0.139
a7 r	8.91	0.085
a7 l	10.17	0.139
a8 r	7.6	0.085
a8 l	9.43	0.139

n/a = insufficient data for statistical testing
