# Supplementary figures and images for: Quantifying Morphological Change in Stage III Lipedema: A 3D Imaging Study of Population Trends and Individual Treatment Courses
Source: J Pers Med. 2025 Nov 1;15(11):525. doi: 10.3390/jpm15110525 (PMC12653540; doi:10.3390/jpm15110525)

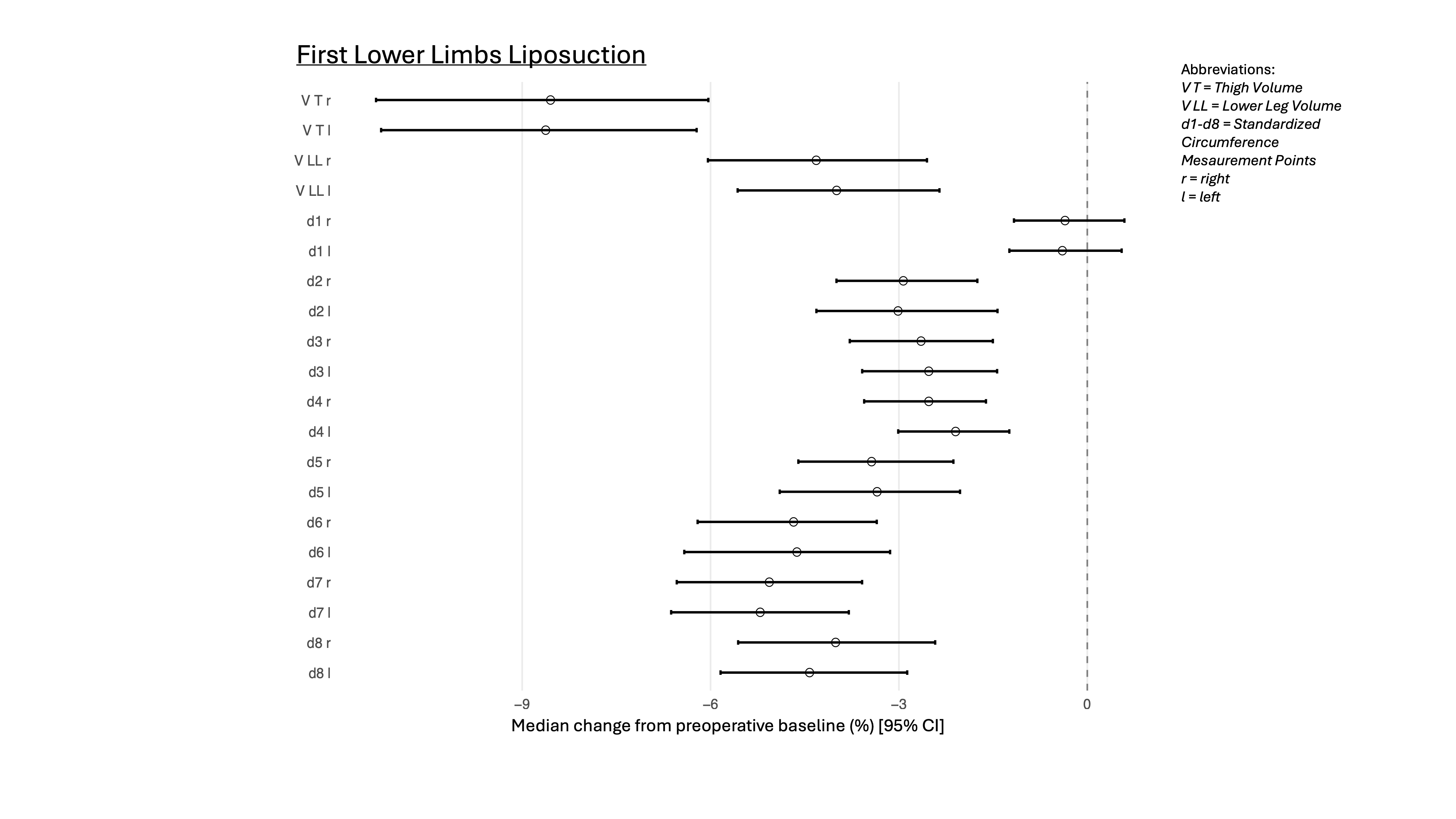

Supplement: Supplementary file 1 [file jpm-15-00525-s001.zip › Figure S1. Lower Limb Liposuction.png]

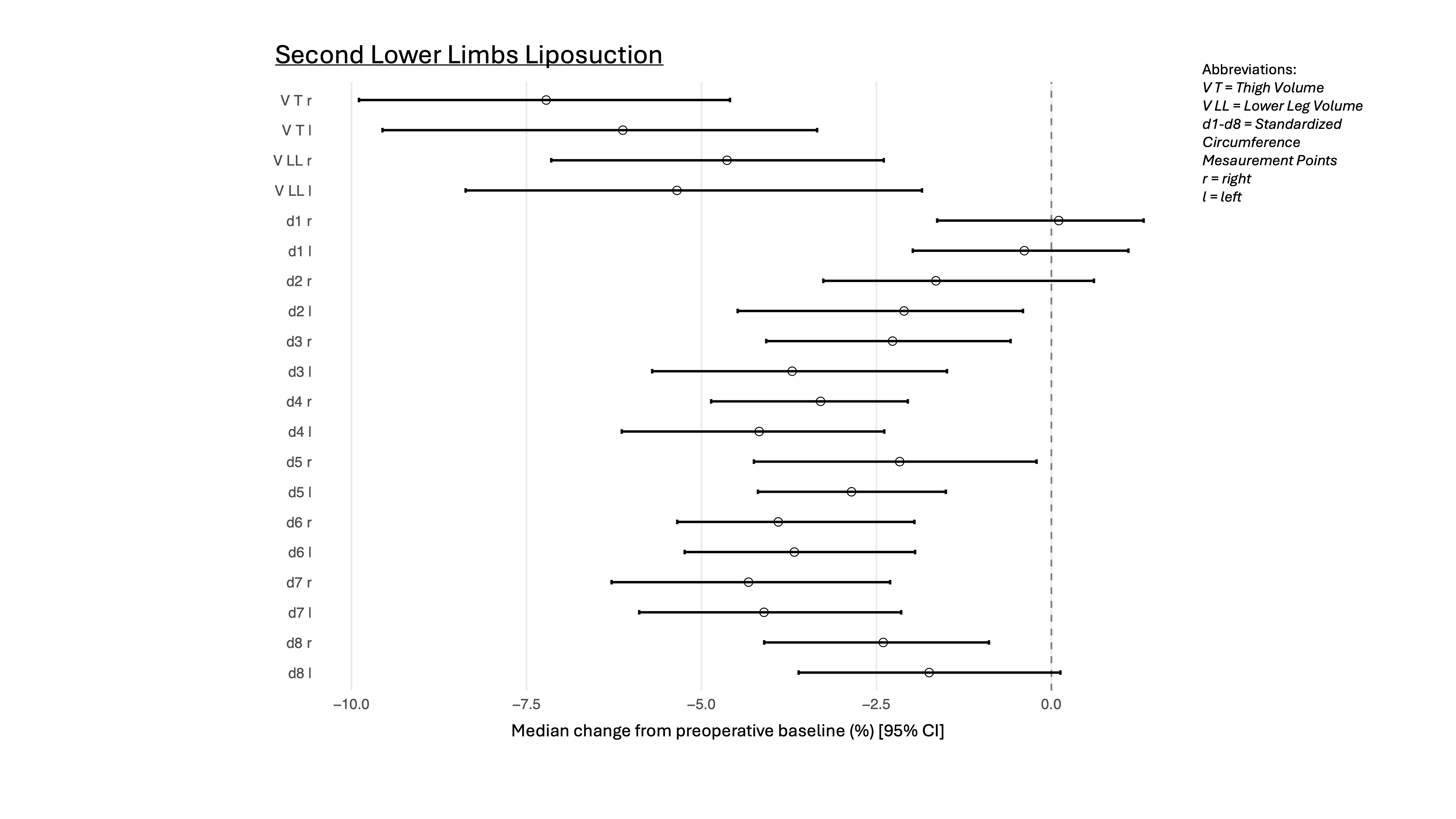

Supplement: Supplementary file 1 [file jpm-15-00525-s001.zip › Figure S2. Lower Limb Liposuction.png]

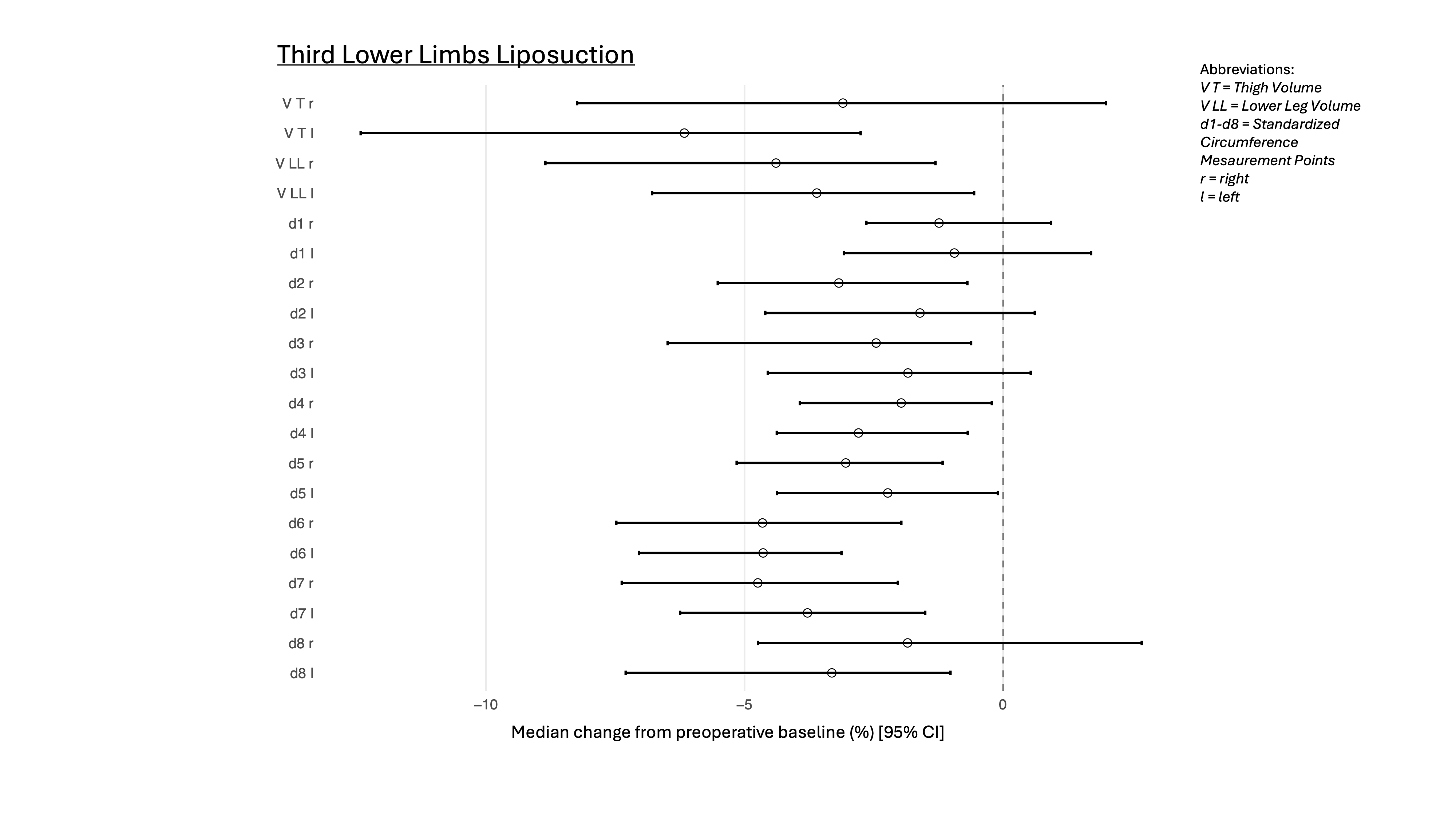

Supplement: Supplementary file 1 [file jpm-15-00525-s001.zip › Figure S3. Lower Limb Liposuction.png]

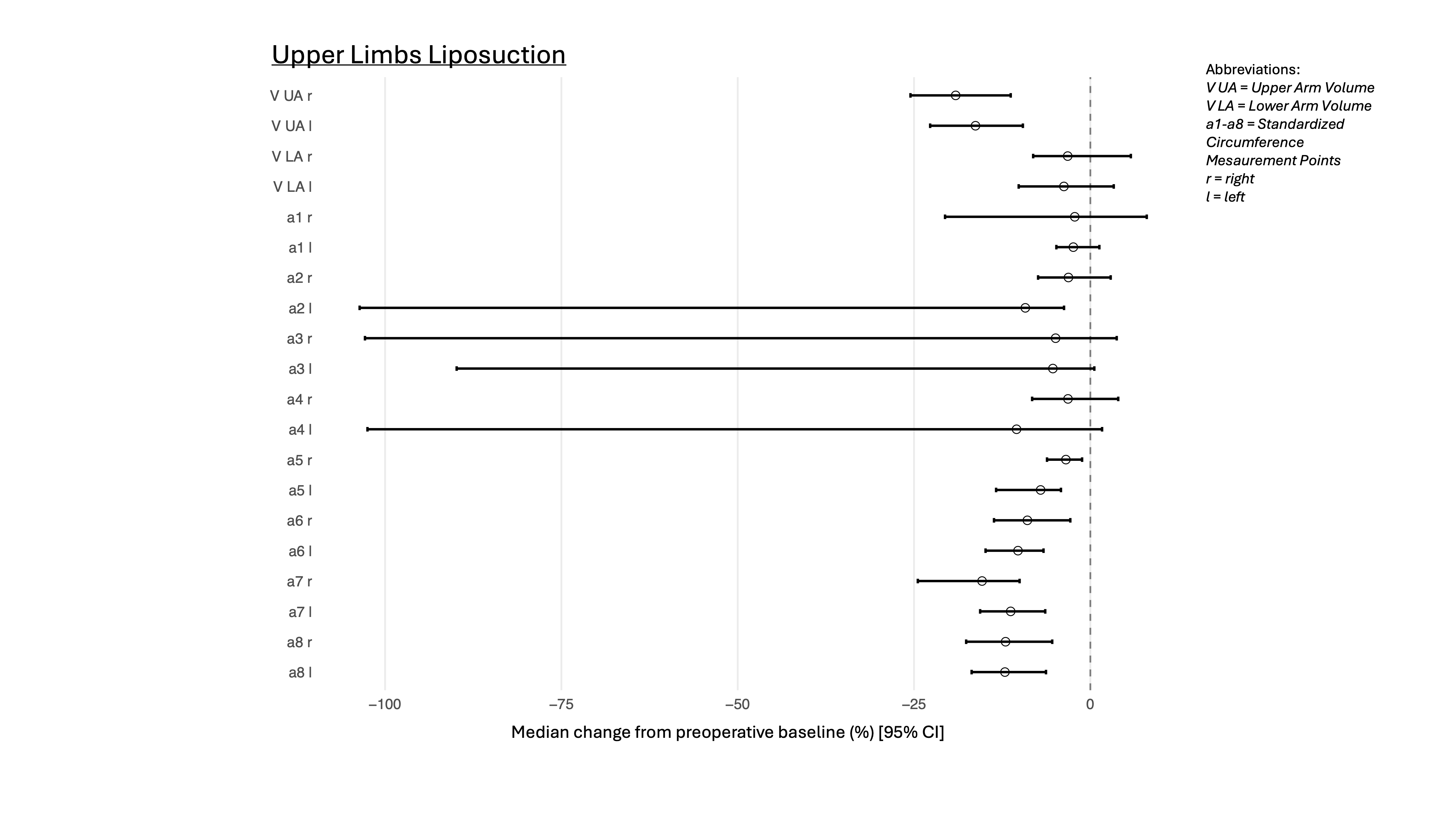

Supplement: Supplementary file 1 [file jpm-15-00525-s001.zip › Figure S4_Upper Limb Liposuction.png]
